# Transcriptome analysis provides insights into copper toxicology in piebald naked carp (*Gymnocypris eckloni*)

**DOI:** 10.1186/s12864-021-07673-4

**Published:** 2021-06-05

**Authors:** Wenjie Jin, Zixuan Li, Fengxia Ran, Shen Huang, Kefan Huo, Jianjuan Li, Qingshuo Han, Guojie Wang, Zhenji Wang, Shenlong Jian, Kemao Li, Changzhong Li

**Affiliations:** 1 College of Eco-Environmental Engineering, Qinghai University, Xining, 810016 China; 2 State Key Laboratory of Plateau Ecology and Agriculture, Qinghai University, No. 521 Ningda Road, Chengbei District, Xining, 810016 China; 3 Fisheries Environmental Monitoring Station, Xining, 810016 China

**Keywords:** Transcriptome, RNA-seq, *Gymnocypris eckloni*, Copper, Toxicology

## Abstract

**Background:**

Copper was used for many years in aquaculture operations as an effective algaecide or a parasite treatment of fish. It is an essential nutrient with numerous functions in organisms, but is toxic at high concentrations. However, the toxicity of copper to fish remains unclear. In this study, we used the piebald naked carp, *Gymnocypris eckloni*, as a model. RNA-seq data from different tissues, including gills, kidney, and liver, were used to investigate the underlying mechanism of copper toxicology in *G. eckloni*.

**Results:**

We compared the transcriptomes from different tissues with different time durations of copper ion treatment. After 72 h copper ion treatment, the number of genes with different expression in gills and liver changed dramatically, but not in kidneys. In KEGG functional enrichment, the pattern of differentially expressed genes (DEGs) was also similar in the gills and liver. The most enriched pathway of DEGs was “Ribosome” in both tissues. Furthermore, we analyzed the expression levels of genes involved in oxidative stress response and protein synthesis using qPCR and RNA-seq data. Our results showed that several genes involved in oxidative stress response were up-regulated both in gills and liver. Up-regulation of these genes indicated that copper treatment caused oxidative stress, which is likely to result in ribosome damage. In addition, our results showed that the expression of *Eef1b2*, a transcription elongation factor, was decreased in the liver under oxidative stress, and the expression of translation initiation factors *Eif4ebp1* and *eIF2α*, and elongation factor *eEF2* was up-regulated. These results supported the idea that oxidative stress inhibits protein synthesis in cells.

**Conclusions:**

Our results indicate that copper exposure caused different responses in different tissues, since the gene expression patterns changed substantially either in the gills or liver, while the effect on the kidney was relatively weak. Furthermore, our results indicated that the expression pattern of the genes involved in the ribosome, which is a complex molecular machine orchestrating protein synthesis in the cell, together with translation initiation factor and elongation factors, were affected by copper exposure both in the gills and liver of piebald naked carp. This result leads us to speculate that the downregulation of global protein synthesis is an acute response strategy of fish to metal-induced oxidative stress. Moreover, we speculate that this strategy not only exists in the selective translation of proteins but also exists in the specific translation of functional proteins in tissues and cells.

**Supplementary Information:**

The online version contains supplementary material available at 10.1186/s12864-021-07673-4.

## Background

Increasing global contamination of aquatic systems is a critical environmental problem. In addition to cadmium, copper is one of the most common pollutants in water. Copper is a basic nutrient with multiple functions in living organisms [1], but it is toxic if its concentration is high [2–4]. It has been used for many years as an effective algaecide or a parasite treatment of fish in aquaculture operations. However, the ambient copper can be absorbed by the fish through the gills, skin, and gastrointestinal tract and transferred through blood to the internal organs [5, 6]. The accumulation of copper causes damage to the homeostasis of essential metals in different organs. Oxidative stress is one of the known mechanisms of copper toxicity to fish [5, 6].

Oxidative stress can be caused by metal ions in model organisms, and biochemical ways to prevent oxidative damage are particularly significant in toxicology. Some fish species, such as zebrafish (*Danio rerio*), goldfish (*Carassius auratus*), and brown trout (*Salmo trutta*), have been well studied as models of the effects of environmental stresses and metal pollutants on oxidative stress and antioxidant resistance in freshwater fish [7–9]. In previous studies, the effects of metal contamination on fish were investigated by measuring the activity of superoxide dismutase (SOD), which is involved in the antioxidant response, and glucose-6-phosphate dehydrogenase (G6PDH) activity which participates in energy accumulation but also in anti-oxidant responses [10]. RNA-seq is an effective method for comprehensively identifying the molecular pathways affected by metal ion exposure, but so far, few studies have been conducted using the technique to study metal toxicology in fish [7, 11], especially many aspects of fish copper poisoning, which remain unclear.

In this study, we use the piebald naked carp (*Gymnocypris eckloni*) as a model. It is a representative species of the subfamily Schizothoracinae, and is the dominant fish in the upper reaches of the Yellow River at an elevation of 3000 m [12, 13]. *G. eckloni* is economically valuable due to its high production capacity. As mentioned above, copper is added to commercial aquaculture or exists in metal-polluted water. However, the effect of copper on the physiological function of different tissues in *G. eckloni* has not been evaluated, but is an important issue in food safety and aquatic toxicology.

In the present study, we used RNA-seq to investigate the response of different *G. eckloni* tissues to a copper concentration of 0.01 mg/L. This concentration is ecologically relevant since it is comparable to those found in water or used in commercial aquaculture [14–16]. Transcriptome data from three different tissues – gills, liver, and kidney – were used to investigate the response to copper exposure. We compared transcriptome changes in these tissues to assess the effects of copper ions on different tissues. In addition, by analyzing the changes in gene expression levels related to oxidative stress and the protein synthesis pathway, we proposed the potential molecular mechanism of copper toxicity in the piebald naked carp.

## Results

### RNA-seq data and de novo transcriptome assembly

To systematically investigate the transcriptome dynamics of the gills, liver, and kidney at different time points after copper ion treatment, we obtained 36 transcriptome samples. After removing low-quality sequences and trimming adapter sequences, 7.18–9.36 Gb of 125-bp paired-end clean reads were generated from each library. The total counts of clean reads were between 47,873,998 and 62,386,764.

Since there is no published genome of this species or a closely related species, we assembled the transcripts de novo and used this assembly as a reference. De novo assembly using all clean reads identified 704,405 transcript fragments, ranging in length from 201 to 23,517 bp, with an average length of 774 bp. The N50 (the length of the contig at 50% of the assembly arranged in descending order according to contig length) and N90 (the length of the contig at 90% of the assembly arranged in descending order according to contig length) values of the obtained transcripts were 1265 and 298 bp, respectively. In further assembly analysis, 486,221 gene fragments were identified across all transcripts, with an average length of 994 bp. The N50 and N90 values of the obtained gene fragments were 1480 and 438 bp, respectively.

The number of transcripts obtained in this work is similar to many published transcriptome assemblies [13, 17–21], including de novo transcriptome assemblies in several teleost species [13, 20, 21]. This indicates that our assembly quality is reliable to be used for subsequent transcriptome data analysis.

### Gene functional annotation

All 486,221 gene fragments were annotated against seven public databases: NCBI non-redundant protein sequences (Nr), NCBI non-redundant nucleotide sequences (Nt), Protein family (Pfam), Clusters of Orthologous Groups of proteins/eukaryotic Ortholog Groups (KOG/COG), Swiss-Prot, Kyoto Encyclopedia of Genes and Genomes (KEGG), and Gene Ontology (GO). A total of 152,017 gene fragments were annotated against the Nr protein database (31.26%). The most common species with high sequence similarity in the Nr database was *Danio rerio* (64.8%), followed by *Astyanax mexicanus* (5.7%), *Clupea harengus* (2.8%), *Ichthyophthirius multifiliis* (2.1%), and *Oncorhynchus mykiss* (2.0%). Most of these species are teleost fish, suggesting the gene fragments used in this study were correctly assembled and annotated.

**Fig. 1:**
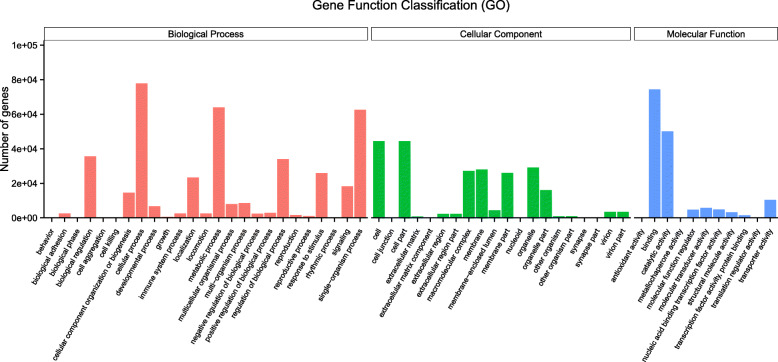
A total of 56 functional groups were identified by GO annotation under the three categories (Biology Process, Cellular Component, and Molecular Function)

After GO annotation, the predicted *G. eckloni* genes were further cataloged in the next subdirectory under the three categories (Biology Process, Cellular Component, and Molecular Function). A total of 56 functional groups are displayed in Fig. 1.

**Fig. 2:**
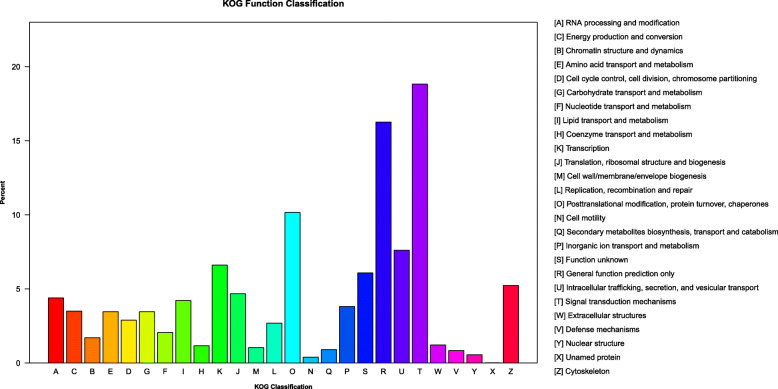
Using the KOG database, a total of 55,116 genes were annotated, which were categorized into 26 groups. The vertical axis represents the proportion of the number of gene fragments in each functional category

KOG annotation assigned the 55,116 genes into 26 groups, which are shown in Fig. 2. Among these categories, the most sequences were assigned to “Signal transduction mechanisms” (10,376 genes), followed by “General function prediction only” (8966 genes), “Posttranslational modification, protein turnover, chaperones” (5602 genes), “Intracellular trafficking, secretion, and vesicular transport” (4198 genes), and “Transcription” (3644 genes).

**Fig. 3:**
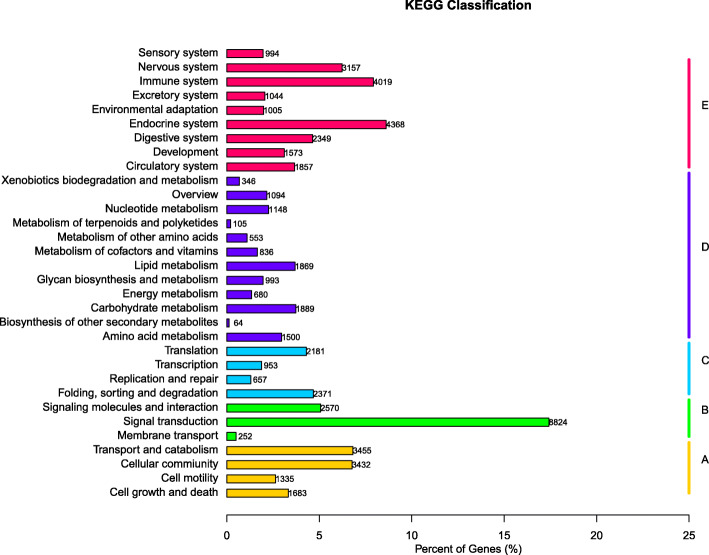
Results of KEGG annotation. **a**, Cellular Processes (9905 genes); **b**, Environmental Information Processing (11,646 genes); **c**, Genetic Information Processing (6162 genes); **d**, Metabolism (11,077 genes); and **e**, Organismal Systems (20,366 genes)

A total of 50,669 genes were annotated using the KEGG database (Fig. 3). These genes were further divided into five groups: A, Cellular Processes (9905 genes); B, Environmental Information Processing (11,646 genes); C, Genetic Information Processing (6162 genes); D, Metabolism (11,077 genes); and E, Organismal Systems (20,366 genes).

### Differentially expressed genes and functional enrichment

**Fig. 4:**
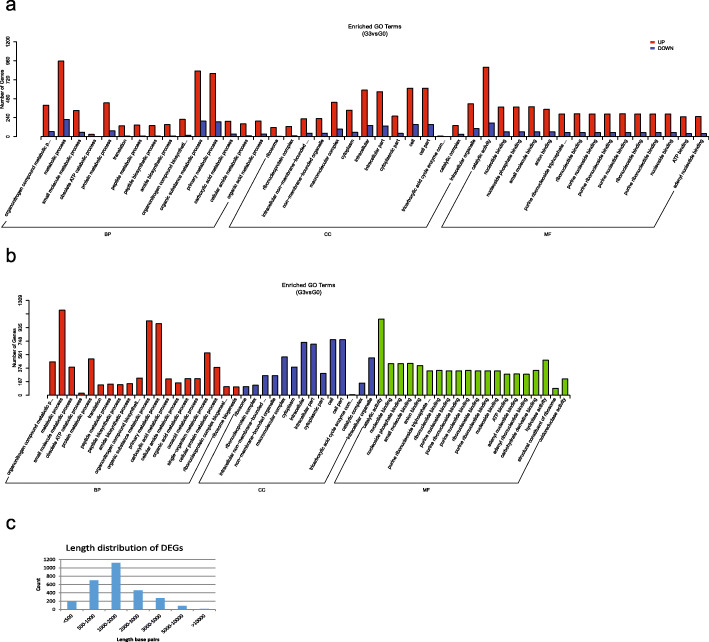
The differentially expressed genes (**a**), functional enrichment (**b**), and length distribution (**c**) in the gills after 72 h of copper ion treatment compared with control

**Fig. 5:**
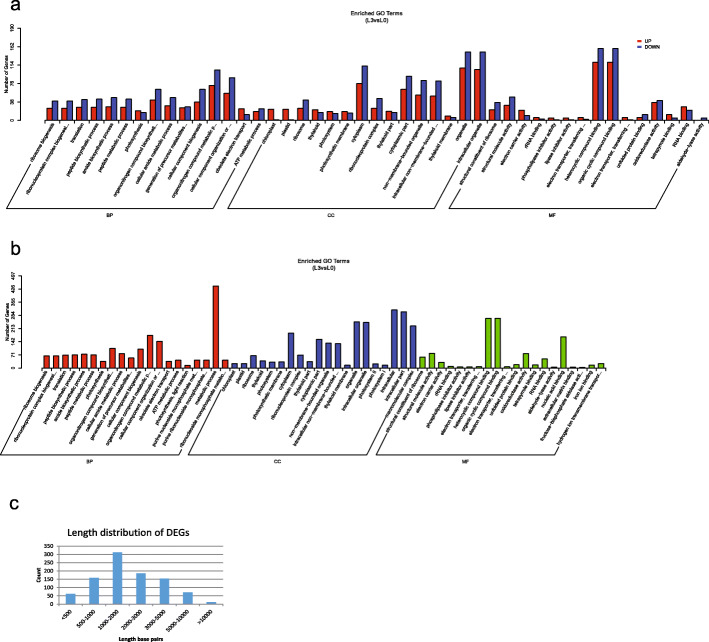
The differentially expressed genes (**a**), functional enrichment (**b**), and length distribution (**c**) in the liver after 72 h of copper ion treatment compared with control

The responses of different tissues of the piebald naked carp to the copper ion treatment were different. In gills, after 6 h of metal ion treatment, 410 and 361 genes were respectively up- and down-regulated. After 36 h of treatment, the numbers of up and down-regulated genes were 327 and 324, respectively. The number of differentially expressed genes (DEGs) was abruptly increased after 72 h of treatment; 2238 and 609 genes were up- and down-regulated, respectively (Fig. 4a). The trend of gene expression patterns in the liver was similar to the gills. The period with the most differences in gene expression level occurred after 72 h of metal ion treatment, with 401 and 555 genes up- and down-regulated, respectively. The respective numbers were 285 and 246 after 6 h, and 325 and 553 after 36 h (Fig. 5a). The numbers of DEGs were relatively constant in different periods in the kidney. After 6 h of copper ion treatment, 422 and 326 genes were up- and down-regulated, respectively. The respective numbers were 332 and 357 after 36 h, and 295 and 255 after 72 h.

To further explore the mechanism of damage of metal ions to organs and the pathways of responses of the organs, we analyzed the functional enrichment of the DEGs. In GO functional enrichment in gills and liver, the most DEGs were enriched in “metabolic process” (Fig. 4b and 5b) under the Biological Process category. In the gills, the DEGs were enriched in the terms “organic substance metabolic process”, “primary metabolic process” under the Biological Process category; “cell” and “cell part” under the Cellular Component category; and “catalytic activity” under the Molecular Function category. In the liver, the DEGs were enriched in the terms “organonitrogen compound metabolic process”, “cellular component biogenesis” under the Biological Process category; “intracellular” and “intracellular part” under the Cellular Component category; and “heterocyclic compound binding” and “organic cyclic compound binding” under the Molecular Function category.

De novo transcriptome assembly often results in a large number of short contigs. To evaluate the potential impact of short contigs on the identification of DEGs, we plotted the length distribution of DEGs (Fig. 4c and 5c). The results indicated that the proportion of short contigs (< 500 bp) in the DEGs was minor (6.81% in gills and 6.49% in the liver).

**Fig. 6:**
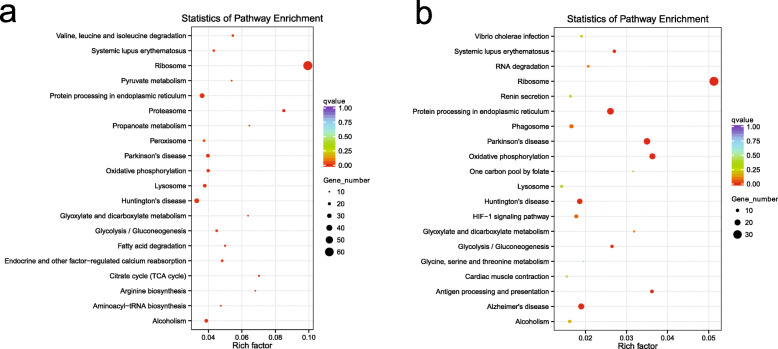
KEGG enrichment of DEGs in gills (**a**) and livers (**b**). The DEGs are from the 72 h copper treatment group vs. the control group

In KEGG functional enrichment, the pattern of DEGs was also similar in the gills and liver. The most enriched pathway of DEGs was “Ribosome” in both tissues. The “Protein processing in endoplasmic reticulum”, “Oxidative phosphorylation”, “Lysosome”, and “Glycolysis/Gluconeogenesis” were also enriched in both tissues (Supplementary file 1 and 2) (Fig. 6).

### Genes involved in oxidative stress and protein synthesis after copper exposure

**Fig. 7:**
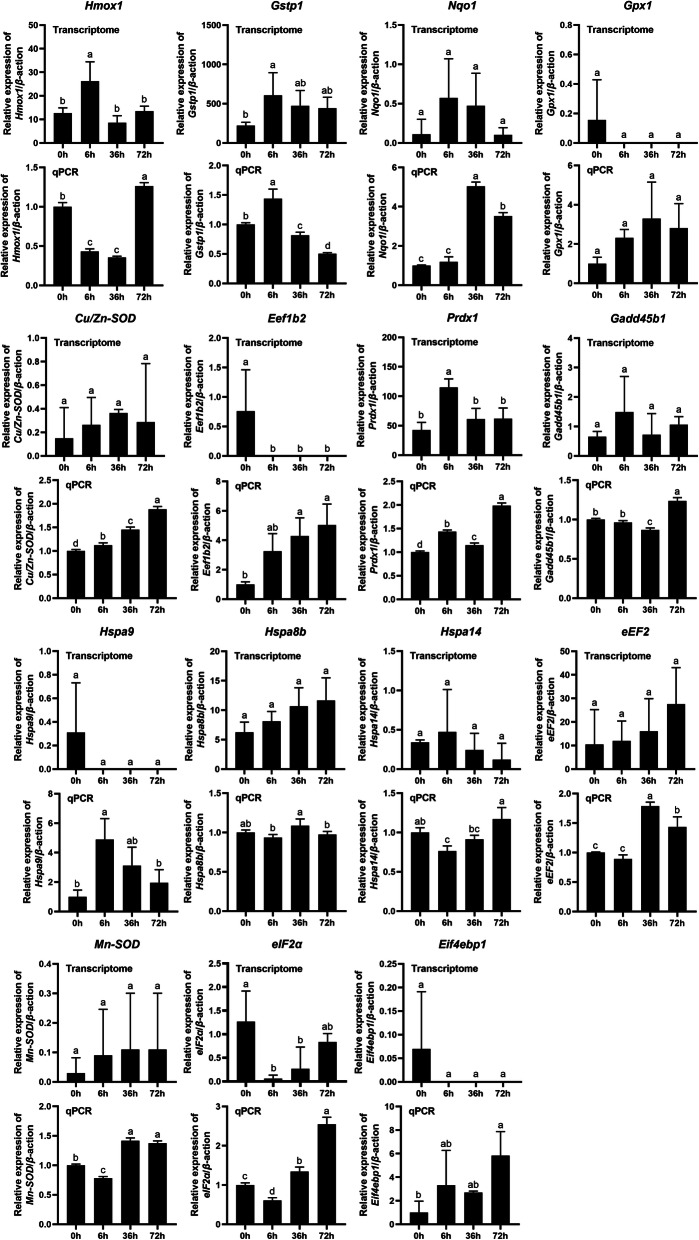
Expression levels of genes involved in oxidative stress and protein synthesis in gills after copper ion stress. The vertical axis represents the relative expression of these genes, the horizontal axis is 0 h, 6 h, 36 h, and 72 h of copper treatment

**Fig. 8:**
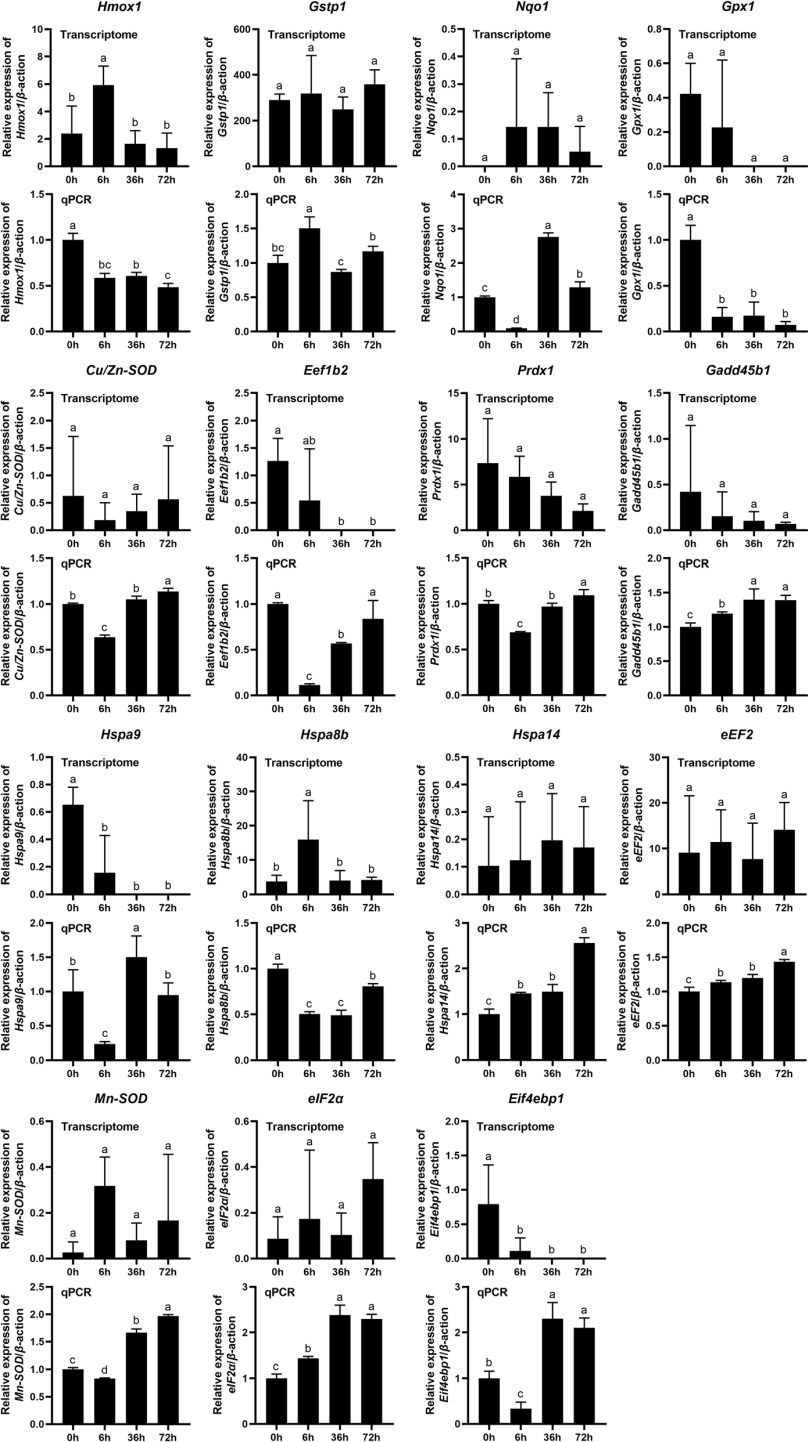
Expression levels of genes involved in oxidative stress and protein synthesis in liver after copper ion stress. The vertical axis represents the relative expression of these genes, the horizontal axis is 0 h, 6 h, 36 h, and 72 h of copper treatment

The DEGs of both gills and liver were mostly enriched in “Ribosome” and oxidative stress response in the copper treated group as compared with control. Therefore, the expression levels of genes involved in oxidative stress response and protein synthesis were measured using qPCR. Our results showed that the expression levels of these genes displayed different patterns after copper treatment. Among the genes involved in oxidative stress, the expression of *Hmox1* (Cluster-47,180.157989), Nqo1 (Cluster-47,180.117564), *Gpx1* (Cluster-47,774.0), *Cu/Zn-SOD* (Cluster-47,180.196300), *Prdx1* (Cluster-47,180.233418), *Gadd45b1* (Cluster-47,180.146985), *Hspa9* (Cluster-47,180.10385), *Hspa14* (Cluster-47,180.185583), and *Mn-SOD* (Cluster-47,180.180330) was greatly up-regulated in the gills (Fig. 7), while the expression of *Nqo1* (Cluster-47,180.117564), *Cu/Zn-SOD* (Cluster-47,180.117564), *Prdx1* (Cluster-47,180.233418), *Gadd45b1* (Cluster-47,180.233418), *Hspa14* (Cluster-47,180.185583), and *Mn-SOD* (Cluster-47,180.180330) was greatly up-regulated in the liver (Fig. 8). Among the genes involved in protein synthesis, the expression levels of translation initiation factors *Eif4ebp1* (Cluster-47,180.374620) and *eIF2α* (Cluster-47,180.297183), and elongation factors *eEF2* (Cluster-47,180.277438) and *Eef1b2* (Cluster-47,453.0), were up-regulated (Figs. 7 and 8). In addition, the expression of *Hmox1* (Cluster-47,180.157989), *Gpx1* (Cluster-47,774.0), *Eef1b2* (Cluster-47,453.0), and *Hspa8b* (Cluster-47,180.176206) was down-regulated in the liver (Fig. 8). In summary, it can be seen that some transcriptome data showed large differences among duplicate samples, but there was general agreement between transcriptome results for whole heavy metal stress and qPCR validation tests, and transcripts basically represented true expression differences.

## Discussion

### Different tissues in fish respond differently to copper exposure

Among the major pollutants in the environment, metal ions pose a serious threat to aquatic organisms due to their unique bioaccumulation and toxicity. When these harmful substances are absorbed by fish, they damage many organs. Cadmium (Cd), for example, is a toxic heavy metal that adversely affects the survival and growth of aquatic organisms [22, 23]. Thus, it is a model oxidative stressor to study the mechanism of chemical toxicity. A recent study found that this toxic substance can lead to a liver malfunction in zebrafish as indicated by RNA-seq data [7]. Also, this heavy metal could elicit oxidative stress during zebrafish larval development [24]. However, in a previous study using yellow perch (*Perca flavescens*) as an experimental model, there was no significant difference in the accumulation of renal metals in Cd-exposed fish compared to controls [10].

Therefore, in this study, we investigated the response of different tissues to exposure to copper. To this end, RNA-seq data were analyzed in three different tissues: gills, liver and kidney. After 72 h of copper ion treatment, there were acute changes in the numbers of DEGs in the gills and liver, but not the kidney. This result indicates that copper exposure caused different responses, since the gene expression patterns changed substantially in the gills and liver, while the effect on the kidney was relatively weak, which is consistent with a previous study (Supplementary Table S2) [10].

### Pathway of metal-induced response in gills and liver

Fish gills are particularly vulnerable to many water-borne pollutants, including heavy metals and chemically toxic substances. These pollutants often cause considerable ultrastructural damage, including epithelial cell proliferation, chloride cell necrosis, lamellar cell swelling and fusion, and mucous cell hyperplasia [25, 26]. In addition, the liver tissue of fish is also vulnerable to environmental stress, such as chemical pollutants and heavy metal ions [27, 28]. Therefore, we explored the response of gills and liver of fish to copper exposure.

From the KEGG enrichment, the differentially expressed genes (DEGs) in both gills and liver were enriched in “Ribosome” (Fig. 6). The ribosome is a complex ribonucleoprotein-based molecular apparatus that that is responsible for the synthesis of proteins within cells. Ribosome proteins and RNA of ribosomes are affected by reactive oxygen radicals, which may impair the homeostasis and stability of cells and even lead to the complete loss of ribosomal function [29]. The binding of divalent metal ions can cause chemical changes in biomacromolecules, either through metal ions directly reacting with the bound molecules or by promoting the local generation of ROS [29]. For example, combining Pb^2+^ with purified *E. coli* ribosomes can results in site-specific cleavages in rRNA [30]. ROS can perform essential biochemical and signaling functions or cause damage to cell components, including ribosomes [31]. In order to minimize damage caused by ROS, cells have developed various defense enzymes that modulate the stability of the system in an optimal or tolerable range. However, once the load of reactive oxygen species exceeds the capacity of the cellular antioxidant system, the organelles in the cells may be damaged and many cell functions may suffer.

Oxidized transition metals such as copper ions play a special role in the stability and stability of ribosomes. Copper is thought to be a transitional metal that promotes oxidative damage of nucleic acids and proteins [32]. In this study, we found that DEGs in copper treated fish gills and liver compared with control were enriched in “Ribosome”. In addition, we evaluated expressional levels of genes involved in oxidative stress response by qPCR. Our results indicate that the expression of *Hmox1* (Cluster-47,180.157989), Nqo1 (Cluster-47,180.117564), *Gpx1* (Cluster-47,774.0), *Cu/Zn-SOD* (Cluster-47,180.196300), *Prdx1* (Cluster-47,180.233418), *Gadd45b1* (Cluster-47,180.146985), *Hspa9* (Cluster-47,180.10385), *Hspa14* (Cluster-47,180.185583), and *Mn-SOD* (Cluster-47,180.180330) was greatly up-regulated in the gills, while the expression of *Nqo1* (Cluster-47,180.117564), *Cu/Zn-SOD* (Cluster-47,180.117564), *Prdx1* (Cluster-47,180.233418), *Gadd45b1* (Cluster-47,180.233418), *Hspa14* (Cluster-47,180.185583), and *Mn-SOD* (Cluster-47,180.180330) was greatly up-regulated in the liver (Fig. 7). In previous study, expressional levels of *Hmox1*, *Nqo1*, *Gpx1*, *Cu/Zn-SOD*, *Prdx1*, *Gadd45b1*, *Hspa9*, *Hspa14*, and *Mn-SOD* were used as indicators to detect oxidative stress in zebrafish larvae [26]. Upward regulation of these genes in the gills and liver suggested that copper treatment induced oxidative stress, which may lead to ribosome damage [29–31]. Therefore, our results support the notion that divalent metal ions can lead to damage of intracellular organelles [31], since gene expression patterns related to ribosome function were affected by copper exposure.

The biological function of ribosomes is to organize protein synthesis in cells. Protein synthesis is a basic, energy-consuming process for all living things. Cells often respond to adverse stimuli through coordinated changes in gene expression [33]. It is noteworthy that some genes involved in regulating cell growth and proliferation, differentiation, and apoptosis use other strategies for translation. Exposure to certain metals, such as hydrargyrum, copper, and cadmium, may lead to the down-regulation of global protein synthesis in some marine organisms [34, 35]. The effect of oxidative stress on protein synthesis is a basic problem in biomedical research. Many studies have shown that oxidative stress inhibits protein synthesis in cells [36, 37]. But little is known about the mechanism of the process. Numerous studies suggested that the regulatory mechanism of protein synthesis involves transcriptional initiation, elongation, and termination [35–38]. In the translational responses to oxidative stress, transcription initiation was inhibited by the initiator, eIF2a, and 4E-BPs phosphorylation [35]. Furthermore, elongation factors were also sensitive to oxidative stress. In *Saccharomyces cerevisiae*, after exposure to H_2_O_2_ and cadmium, the elongation factor eEF1Bα, eEF2 and eEF3 were reduced immediately [38]. Similarly, our results relate to the extended stage of translation regulation: *Eef1b2* (Cluster-47,453.0) expression level was decreased in the liver under oxidative stress. Moreover, *Hmox1* (Cluster-47,180.157989), *Gpx1* (Cluster-47,774.0), and *Hspa8b* (Cluster-47,180.176206) were also decreased in the liver after copper ion stress. Therefore, we speculate that this strategy not only exists in the selective translation of proteins, but is also present in the specific translation of functional proteins in tissues and cells.

## Conclusions

RNA-seq from gills, liver, and kidney of piebald naked carp to investigate the mechanism of copper toxicology. Our results show that the response of different tissues to copper exposure varied substantially. The gene expression patterns changed substantially in the gills and liver, indicating that these tissues are vulnerable to waterborne pollutants. In contrast, the kidney is relatively less sensitive. Furthermore, our results support the notion that divalent metal ions can affect the expression of genes involved in ribosome, and thus inflict damage on cellular components, since genes involved in the ribosome displayed different expression patterns. Also, the translation stage of proteins changes on a large scale during oxidative stress. Copper exposure can lead to a decrease in the synthesis of some proteins by down-regulating the expression level of elongation factors, and increase the translation of other proteins to repair damage and adapt to a stressful environment.

## Methods

### Fish samples and treatment

About 3 years old adult male piebald naked carp (*Gymnocypris eckloni*), weight 100 ± 2.5 g, Length 19 ± 3.4 cm, were obtained from the Fisheries Environmental Monitoring Station, Qinghai Province, China, and brought back to the laboratory in Qinghai University. During the domestication period-two weeks, fish are raised in fresh water at 15 ± 0.5 °C with a composition almost identical to that of rivers in wild habitats. At the end of this period, fish were placed in several 40 L glass aquariums with circulation systems and kept at 15 ± 0.5 °C during a 14 h:10 h light/dark cycle.

After acclimation, naked carp were exposed to metal ion stressors for different time durations. Exposure levels of oxygen and diet are the same as during adaptation. Twelve fish were randomly sampled and divided into four groups, the control group, heavy metal treated groups for 6, 36, and 72 h, and each group contained 3 fish.

CuSO_4_·5H_2_O was purchased from Sigma-Aldrich Chemical Co. (St Louis, MO, USA). The National Drinking Water Quality Standards in the People’s Republic of China (GB5749–2006) (copper ion concentration is not greater than 0.01 mg/L) was adopted to detect a series of biomarkers, which can more realistically reflect the response of aquatic organisms to heavy metal pollutants, and have more practical significance for the early warning of heavy metal pollution in water quality and environment. Besides, the metal ion concentration was chosen based on published works [10, 11]. Test solutions were completely changed every 24 h.

### RNA extraction and transcriptome sequencing

Animal euthanasia was under the approved QHU Laboratory Animal Care and Use Committee protocols. In short, each fish was placed in a separate 2 L container containing 1.5 L of unbuffered 0.1% (100 mg/L) of clove oil until they lost their sense of direction and the gill cover stopped moving. Generally, adult fish of 19 ± 3.4 cm usually need 2–5 min. After 0.01 mg/L Cu^2+^ stressed for 6, 36, and 72 h, the gills, liver, and kidney were dissected and immediately frozen in the liquid nitrogen, stored at − 80 °C until RNA extraction.

Total RNA was extracted using RNAiso Plus (TaKaRa) as described by the manufacturer, and then perform DNase I processing. Degradation and contamination of RNA were detected by 1% agarose gel. In order to check the purity of RNA, the A260/A280 ratios of all RNA samples were measured by using the NanoPhotometer® spectrophotometer (IMPLEN, CA, USA). The RNA integrity was assessed with the RNA integrity number (RIN) values by using RNA Nano 6000 Assay Kit of the Agilent Bioanalyzer 2100 system (Agilent Technologies, CA, USA). Thirty-six separate Illumina sequencing libraries were established with 1.0 μg RNA per sample.

Sequencing libraries for each sample (3 replicates per sample) were prepared with NEBNext® Ultra™ RNA Library Prep Kit for Illumina (NEB, USA) following the manufacturer’s recommendations. An index code was added to assign a sequence to each sample. The sequencing was performed on Illumina HiSeq 2500 instrument at Novogene Bioinformatics Institute, Beijing, China.

### Quality control and de novo transcriptome assembly

The original data in FASTQ format was filtered and processed with a self-written Perl script. In this step, adapter sequences, read containing poly-N, and low-quality reads in raw data were removed to generate clean data. In addition, levels of Q20, Q30, GC content and sequence duplication of clean data were calculated to monitor data quality. All downstream analyses were based on high-quality clean data.

The high-quality short reading of all samples was performed using Trinity software to produce a single de novo transcriptome assembly a single end-to-end transcriptome component [39], in which min_kmer_cov set to 2 and all other parameters set by default. First, all overlapping *k*-mers were extracted from the RNA-seq reads to generate transcript contigs using a greedy extension based on (*k*-1)-mer overlaps. In this step, full-length transcripts for a dominant isoform were generated, but only the unique portions of alternatively spliced transcripts were retained for subsequent analysis. Next, the related contigs were clustered using the raw reads to group transcripts based on shared read support and paired read links, when available. These steps were repeated to connect the contigs until the sequences could no longer be extended. Finally, all plausible transcript sequences, resolving alternatively spliced isoforms and transcripts derived from paralogous genes, were generated by analyzing the paths taken by reads and read pairings in the de Bruijn graph [40]. These sequences were considered to be unigenes.

### Gene functional annotation

Candidate coding regions within transcript sequences were identified using TransDecoder (http://transdecoder.github.io/). The functional annotation of the unigenes was conducted using the BLASTX algorithm of the DIAMOND program against 3 reference databases: the NCBI non-redundant (Nr) protein database, the Clusters of Orthologous Groups of proteins (KOG/COG) database, and the Swiss-Prot database [41]. The functional annotation was also performed using four other databases, including the NCBI non-redundant (Nr) nucleotide database by NCBI blast 2.2.28+, the Protein family (Pfam) database by hmmscan in HMMER 3.0 package, the Kyoto Encyclopedia of Genes and Genomes (KEGG) database by KAAS [42], and the Gene Ontology (GO) database by Blast2GO v2.5 [43].

### Differential expression analysis and functional enrichment

The level of gene expression was measured using the criteria of reads per kb per million mapped reads (RPKM) [44]. Differentially expressed genes (DEGs) were detected using the DESeq software [45]. The *P* values were adjusted using the Benjamini and Hochberg multiple testing for controlling the false discovery rate. The gene with an adjusted *P*-value < 0.05 was assigned as differentially expressed. GO enrichment analysis was performed by the GOseq R package [46], and the gene length deviation was corrected. KEGG enrichment was performed by the KOBAS software [47]. Significance analysis was performed by Fisher’s Exact Test.

### The expression level of genes involved in oxidative stress and protein synthesis

Seven putative Nrf2-dependent antioxidant genes (*hmox1*, *sod1*, *sod2*, *gstp1*, *prdx1*, *gpx1*, *nqo1*), three stress response gene (*Hspa9*, *Hspa8*, *Hspa14*), an inducible DNA damage repair gene (*gadd45bb*), two translation initiation factors (*eIF2α, Eif4ebp1*), and two elongation factors (*EeF1b2, eEF2*) were identified from the gene prediction results [24, 35]. Expression levels of these genes were measured using qPCR and RNA-seq data [48].

### qPCR analysis

To validate the RNA-seq results, qPCR was performed by the following method. The product cDNA was diluted into 50 ng/μL, and 1 μL was used for template. The qRT-PCR reaction mixtures (20 μL) also contained 0.8 μL of each gene specific primer, 10 μL abm®EvaGreen qPCR MasterMix-no dye (abm, Canada) and 7.4 μL RNase-free water. The thermal cycling conditions are as follows: pre-denaturation at 95 °C for 10 min, 40 cycles of denaturation at 95 °C for 30 s, annealing at 60 °C for 1 min, followed by extension at 72 °C for 30 s in Roche LightCycler 480 Real-Time PCR system. The 2^−ΔΔCt^ method was used to calculate the relative gene expression level. The *β-actin* of *Gymnocypris eckloni* was chosen as a reference gene to determine the relative level of the target genes. All qPCR experiments were performed in triplicate (Supplementary file 3) and the gene-specific primers were listed in supplementary table (Table S1).

## Supplementary Information

**Additional file 1.** G3vsG0.DEG enriched KEGG pathway API.

**Additional file 2.** L3vsL0.DEG enriched KEGG pathway API.

**Additional file 3.** qPCR data.

**Additional file 4: Table S1.** List of primers used in qRT-PCR

**Additional file 5: Table S2.** Numbers of transcripts and genes in published papers.

## Data Availability

The datasets supporting the results of this article are available in the NCBI BioProject (https://www.ncbi.nlm.nih.gov/bioproject/PRJNA666236) and NCBI Sequence Read Archive (SRA) repository (https://www.ncbi.nlm.nih.gov/sra/) under accession PRJNA666236.

## Abbreviations

SOD: Superoxide dismutase
G6PDH: Glucose-6-phosphate dehydrogenase
Cd: Cadmium
ROS: Reactive oxygen species
QHU: Qinghai University
RIN: RNA integrity number
