# Supplementary material for: Transcriptome analysis provides insights into copper toxicology in piebald naked carp (Gymnocypris eckloni)
Source: BMC Genomics. 2021 Jun 5;22:416. doi: 10.1186/s12864-021-07673-4 (PMC8178853; doi:10.1186/s12864-021-07673-4)
Supplement: Supplementary file 1 — Additional file 1. G3vsG0.DEG enriched KEGG pathway API. [file 12864_2021_7673_MOESM1_ESM.html]

Pathway Enrichment

  

# The most enriched pathway terms

Statistic method: hypergeometric test

FDR correction method: Benjamini and Hochberg

| Term | Sample number | Background number | P-value | Corrected P-value | UniGenes | KO | Entrez ID | Ensembl ID | Gene name |
| --- | --- | --- | --- | --- | --- | --- | --- | --- | --- |
| Ribosome | 3 | 119 | 0.038593295082 | 0.559671821802 | Cluster-47180.120981 Cluster-47180.172578 Cluster-47180.328525 | K02966 K02882 K02917 | NA | NA | RP-S19e, RPS19 RP-L18Ae, RPL18A RP-L35Ae, RPL35A |
| Arginine biosynthesis | 2 | 50 | 0.0402309180635 | 0.559671821802 | Cluster-47180.206337 Cluster-47180.206339 | K01940 K01940 | NA | NA | argG, ASS1 argG, ASS1 |
| Betalain biosynthesis | 1 | 8 | 0.0538243837398 | 0.559671821802 | Cluster-47180.262120 | K00545 | NA | NA | COMT |
| Alanine, aspartate and glutamate metabolism | 2 | 79 | 0.0879325939475 | 0.559671821802 | Cluster-47180.206337 Cluster-47180.206339 | K01940 K01940 | NA | NA | argG, ASS1 argG, ASS1 |
| Tyrosine metabolism | 2 | 83 | 0.0954217505862 | 0.559671821802 | Cluster-47180.262120 Cluster-47180.169382 | K00545 K00129 | NA | NA | COMT E1.2.1.5 |
| Fructose and mannose metabolism | 2 | 90 | 0.108946690316 | 0.559671821802 | Cluster-47180.146844 Cluster-47180.124421 | K00850 K17497 | NA | NA | pfkA, PFK PMM |
| N-Glycan biosynthesis | 2 | 95 | 0.118902658656 | 0.559671821802 | Cluster-47180.310042 Cluster-47180.270177 | K03846 K07966 | NA | NA | ALG9 B4GALT1 |
| Galactose metabolism | 2 | 95 | 0.118902658656 | 0.559671821802 | Cluster-47180.270177 Cluster-47180.146844 | K07966 K00850 | NA | NA | B4GALT1 pfkA, PFK |
| Phenylalanine metabolism | 1 | 28 | 0.16344435334 | 0.559671821802 | Cluster-47180.169382 | K00129 | NA | NA | E1.2.1.5 |
| Glycolysis / Gluconeogenesis | 2 | 117 | 0.165036109191 | 0.559671821802 | Cluster-47180.146844 Cluster-47180.169382 | K00850 K00129 | NA | NA | pfkA, PFK E1.2.1.5 |
| One carbon pool by folate | 1 | 35 | 0.198780491109 | 0.559671821802 | Cluster-47180.226914 | K11787 | NA | NA | GART |
| Protein processing in endoplasmic reticulum | 4 | 376 | 0.203060561763 | 0.559671821802 | Cluster-47180.337024 Cluster-47180.174006 Cluster-47180.54823 Cluster-47180.183591 | K14009 K14007 K09580 K14011 | NA | NA | BCAP31, BAP31 SEC24 PDIA1, P4HB UBXN6, UBXD1 |
| Glycosphingolipid biosynthesis - lacto and neolacto series | 1 | 42 | 0.232648272633 | 0.559671821802 | Cluster-47180.270177 | K07966 | NA | NA | B4GALT1 |
| Endocytosis | 6 | 685 | 0.246982502688 | 0.559671821802 | Cluster-47180.351087 Cluster-47180.231286 Cluster-47180.251653 Cluster-47180.230601 Cluster-47180.286000 Cluster-47180.245241 | K04646 K04189 K06751 K04679 K12484 K18439 | NA | NA | CLTC CXCR4 MHC1 MADHIP, SARA RAB11FIP1\_2\_5 ARAP1 |
| Inositol phosphate metabolism | 3 | 278 | 0.247063500787 | 0.559671821802 | Cluster-47180.231696 Cluster-47180.193925 Cluster-47180.246271 | K18082 K00922 K01099 | NA | NA | MTMR3\_4, ZFYVE10\_11 PIK3C E3.1.3.36 |
| RNA degradation | 2 | 156 | 0.25227706433 | 0.559671821802 | Cluster-47180.242543 Cluster-47180.146844 | K12580 K00850 | NA | NA | CNOT3, NOT3 pfkA, PFK |
| Proteasome | 1 | 48 | 0.260554660288 | 0.559671821802 | Cluster-47180.198739 | K03039 | NA | NA | PSMD13, RPN9 |
| Pentose phosphate pathway | 1 | 49 | 0.265107705064 | 0.559671821802 | Cluster-47180.146844 | K00850 | NA | NA | pfkA, PFK |
| Histidine metabolism | 1 | 58 | 0.30486427356 | 0.609728547119 | Cluster-47180.169382 | K00129 | NA | NA | E1.2.1.5 |
| beta-Alanine metabolism | 1 | 63 | 0.326029591371 | 0.619456223604 | Cluster-47180.169382 | K00129 | NA | NA | E1.2.1.5 |
| Oxidative phosphorylation | 2 | 202 | 0.356466990001 | 0.645035505715 | Cluster-47180.262205 Cluster-47180.237063 | K00413 K02263 | NA | NA | CYC1, CYT1, petC COX4 |
| Starch and sucrose metabolism | 1 | 94 | 0.443807747638 | 0.700424668894 | Cluster-47180.170895 | K00693 | NA | NA | GYS |
| Amino sugar and nucleotide sugar metabolism | 1 | 96 | 0.450669227806 | 0.700424668894 | Cluster-47180.124421 | K17497 | NA | NA | PMM |
| Other types of O-glycan biosynthesis | 1 | 97 | 0.454068694511 | 0.700424668894 | Cluster-47180.270177 | K07966 | NA | NA | B4GALT1 |
| Base excision repair | 1 | 99 | 0.460805703219 | 0.700424668894 | Cluster-47180.177684 | K03648 | NA | NA | UNG, UDG |
| Ether lipid metabolism | 1 | 116 | 0.51486636983 | 0.752497002059 | Cluster-47180.161971 | K01122 | NA | NA | ENPP2 |
| ABC transporters | 1 | 126 | 0.544139024112 | 0.765825293195 | Cluster-27927.0 | K05677 | NA | NA | ABCD3, PMP70 |
| Sphingolipid metabolism | 1 | 136 | 0.571673535648 | 0.775842655523 | Cluster-47180.201971 | K04716 | NA | NA | SGPP1 |
| Ribosome biogenesis in eukaryotes | 1 | 148 | 0.602563143648 | 0.789565498574 | Cluster-47180.278932 | K11108 | NA | NA | RCL1 |
| Glycerolipid metabolism | 1 | 171 | 0.655770574773 | 0.811695311067 | Cluster-47180.175437 | K13506 | NA | NA | GPAT3\_4, AGPAT9, AGPAT6 |
| Lysine degradation | 1 | 174 | 0.662172490607 | 0.811695311067 | Cluster-47180.214924 | K15588 | NA | NA | NSD1 |
| Ubiquitin mediated proteolysis | 1 | 219 | 0.745265375676 | 0.863441463744 | Cluster-47180.351241 | K07868 | NA | NA | RHOBTB1\_2 |
| Peroxisome | 1 | 231 | 0.763792629082 | 0.863441463744 | Cluster-27927.0 | K05677 | NA | NA | ABCD3, PMP70 |
| Spliceosome | 1 | 237 | 0.772552888613 | 0.863441463744 | Cluster-47180.217810 | K12886 | NA | NA | HNRNPK |
| Glycerophospholipid metabolism | 1 | 286 | 0.833104058093 | 0.904512977359 | Cluster-47180.175437 | K13506 | NA | NA | GPAT3\_4, AGPAT9, AGPAT6 |
| Phagosome | 1 | 326 | 0.87052739355 | 0.905393409929 | Cluster-47180.251653 | K06751 | NA | NA | MHC1 |
| RNA transport | 1 | 340 | 0.881567267563 | 0.905393409929 | Cluster-47180.235071 | K03258 | NA | NA | EIF4B |
| Purine metabolism | 1 | 393 | 0.915586985858 | 0.915586985858 | Cluster-47180.226914 | K11787 | NA | NA | GART |
