# Supplementary material for: Transcriptome analysis provides insights into copper toxicology in piebald naked carp (Gymnocypris eckloni)
Source: BMC Genomics. 2021 Jun 5;22:416. doi: 10.1186/s12864-021-07673-4 (PMC8178853; doi:10.1186/s12864-021-07673-4)
Supplement: Supplementary file 2 — Additional file 2. L3vsL0.DEG enriched KEGG pathway API. [file 12864_2021_7673_MOESM2_ESM.html]

Pathway Enrichment

  

# The most enriched pathway terms

Statistic method: hypergeometric test

FDR correction method: Benjamini and Hochberg

| Term | Sample number | Background number | P-value | Corrected P-value | UniGenes | KO | Entrez ID | Ensembl ID | Gene name |
| --- | --- | --- | --- | --- | --- | --- | --- | --- | --- |
| Protein processing in endoplasmic reticulum | 12 | 376 | 2.5661111407e-06 | 9.49461122059e-05 | Cluster-47180.284289 Cluster-47180.242706 Cluster-47180.206640 Cluster-47180.273523 Cluster-47180.294880 Cluster-47180.273521 Cluster-47180.163694 Cluster-47180.176555 Cluster-47180.252354 Cluster-47180.337024 Cluster-47180.183591 Cluster-47180.254559 | K10080 K13249 K09054 K10976 K13989 K10976 K13999 K12275 K08057 K14009 K14011 K09584 | NA | NA | LMAN1, ERGIC53 SSR1 ATF6A ERO1LB DERL2\_3 ERO1LB CKAP4, CLIMP63 SEC62 CALR BCAP31, BAP31 UBXN6, UBXD1 PDIA6, TXNDC7 |
| One carbon pool by folate | 2 | 35 | 0.0207458810736 | 0.269048701176 | Cluster-47180.132484 Cluster-47180.226913 | K13990 K11787 | NA | NA | FTCD GART |
| Protein export | 2 | 36 | 0.0218147595548 | 0.269048701176 | Cluster-47180.176555 Cluster-47180.269673 | K12275 K12272 | NA | NA | SEC62 SRPRB, SRP102 |
| Flavone and flavonol biosynthesis | 1 | 6 | 0.0414184217866 | 0.383120401526 | Cluster-47180.308258 | K01195 | NA | NA | uidA, GUSB |
| Betalain biosynthesis | 1 | 8 | 0.0529393722509 | 0.391751354657 | Cluster-47180.262120 | K00545 | NA | NA | COMT |
| Phagosome | 4 | 326 | 0.136856690754 | 0.784189054317 | Cluster-47180.247553 Cluster-47180.252354 Cluster-47180.232039 Cluster-47180.245507 | K00921 K08057 K06503 K02154 | NA | NA | PIKFYVE, FAB1 CALR TFRC, CD71 ATPeV0A, ATP6N |
| Pantothenate and CoA biosynthesis | 1 | 32 | 0.181013613479 | 0.784189054317 | Cluster-47180.352268 | K01464 | NA | NA | DPYS, dht, hydA |
| Terpenoid backbone biosynthesis | 1 | 40 | 0.219796657807 | 0.784189054317 | Cluster-47180.295691 | K00787 | NA | NA | FDPS |
| Ribosome biogenesis in eukaryotes | 2 | 148 | 0.22835860989 | 0.784189054317 | Cluster-47180.278932 Cluster-47180.283951 | K11108 K14571 | NA | NA | RCL1 RIX7, NVL |
| Glycosaminoglycan degradation | 1 | 45 | 0.243113421834 | 0.784189054317 | Cluster-47180.308258 | K01195 | NA | NA | uidA, GUSB |
| Glycerolipid metabolism | 2 | 171 | 0.279826664046 | 0.784189054317 | Cluster-47180.147665 Cluster-47180.222935 | K15728 K15728 | NA | NA | LPIN LPIN |
| Glyoxylate and dicarboxylate metabolism | 1 | 58 | 0.300579588242 | 0.784189054317 | Cluster-47180.356139 | K18123 | NA | NA | HOGA1 |
| Histidine metabolism | 1 | 58 | 0.300579588242 | 0.784189054317 | Cluster-47180.132484 | K13990 | NA | NA | FTCD |
| beta-Alanine metabolism | 1 | 63 | 0.321520956882 | 0.784189054317 | Cluster-47180.352268 | K01464 | NA | NA | DPYS, dht, hydA |
| Pentose and glucuronate interconversions | 1 | 72 | 0.357673809563 | 0.784189054317 | Cluster-47180.308258 | K01195 | NA | NA | uidA, GUSB |
| Porphyrin and chlorophyll metabolism | 1 | 77 | 0.376933554855 | 0.784189054317 | Cluster-47180.308258 | K01195 | NA | NA | uidA, GUSB |
| Tyrosine metabolism | 1 | 83 | 0.399297171612 | 0.784189054317 | Cluster-47180.262120 | K00545 | NA | NA | COMT |
| Glycine, serine and threonine metabolism | 1 | 86 | 0.410181241989 | 0.784189054317 | Cluster-47180.163282 | K00306 | NA | NA | PIPOX |
| Fructose and mannose metabolism | 1 | 90 | 0.424392351897 | 0.784189054317 | Cluster-47180.124421 | K17497 | NA | NA | PMM |
| Starch and sucrose metabolism | 1 | 94 | 0.438266822305 | 0.784189054317 | Cluster-47180.308258 | K01195 | NA | NA | uidA, GUSB |
| Amino sugar and nucleotide sugar metabolism | 1 | 96 | 0.445080274072 | 0.784189054317 | Cluster-47180.124421 | K17497 | NA | NA | PMM |
| Glycerophospholipid metabolism | 2 | 286 | 0.521493820986 | 0.834335016135 | Cluster-47180.147665 Cluster-47180.222935 | K15728 K15728 | NA | NA | LPIN LPIN |
| Arginine and proline metabolism | 1 | 121 | 0.52369296266 | 0.834335016135 | Cluster-47180.356139 | K18123 | NA | NA | HOGA1 |
| Sphingolipid metabolism | 1 | 136 | 0.565492713485 | 0.834335016135 | Cluster-47180.164862 | K04710 | NA | NA | CERS |
| Arachidonic acid metabolism | 1 | 143 | 0.58374436996 | 0.834335016135 | Cluster-47180.253248 | K00458 | NA | NA | ALOX12 |
| mRNA surveillance pathway | 1 | 144 | 0.586289470798 | 0.834335016135 | Cluster-47180.256329 | K12875 | NA | NA | ACIN1, ACINUS |
| RNA degradation | 1 | 156 | 0.615663148133 | 0.84368653633 | Cluster-47180.242543 | K12580 | NA | NA | CNOT3, NOT3 |
| Lysine degradation | 1 | 174 | 0.655920395203 | 0.859734051465 | Cluster-47180.163282 | K00306 | NA | NA | PIPOX |
| Oxidative phosphorylation | 1 | 202 | 0.710447726265 | 0.859734051465 | Cluster-47180.245507 | K02154 | NA | NA | ATPeV0A, ATP6N |
| Pyrimidine metabolism | 1 | 208 | 0.720976301407 | 0.859734051465 | Cluster-47180.352268 | K01464 | NA | NA | DPYS, dht, hydA |
| Ubiquitin mediated proteolysis | 1 | 219 | 0.739310469996 | 0.859734051465 | Cluster-47180.216202 | K10615 | NA | NA | HERC4 |
| Peroxisome | 1 | 231 | 0.757962209882 | 0.859734051465 | Cluster-47180.163282 | K00306 | NA | NA | PIPOX |
| Spliceosome | 1 | 237 | 0.766789829685 | 0.859734051465 | Cluster-47180.256329 | K12875 | NA | NA | ACIN1, ACINUS |
| Endocytosis | 3 | 685 | 0.793803359182 | 0.863844832051 | Cluster-47180.296184 Cluster-47180.221554 Cluster-47180.232039 | K18440 K12495 K06503 | NA | NA | ARAP2 IQSEC TFRC, CD71 |
| Inositol phosphate metabolism | 1 | 278 | 0.81919542754 | 0.866006594828 | Cluster-47180.247553 | K00921 | NA | NA | PIKFYVE, FAB1 |
| RNA transport | 1 | 340 | 0.877220059623 | 0.901587283501 | Cluster-47180.256329 | K12875 | NA | NA | ACIN1, ACINUS |
| Purine metabolism | 1 | 393 | 0.911986387442 | 0.911986387442 | Cluster-47180.226913 | K11787 | NA | NA | GART |
