# Supplementary material for: Transcriptome analysis provides insights into copper toxicology in piebald naked carp (Gymnocypris eckloni)
Source: BMC Genomics. 2021 Jun 5;22:416. doi: 10.1186/s12864-021-07673-4 (PMC8178853; doi:10.1186/s12864-021-07673-4)
Supplement: Supplementary file 4 — Additional file 4: Table S1. List of primers used in qRT-PCR [file 12864_2021_7673_MOESM4_ESM.docx]

Table S1 List of primers used in qRT-PCR

| Genes | Forward primer (5' to 3') | Reverse primer (5' to 3') |
| --- | --- | --- |
| *Eif4ebp1* | CACGCTCTTCAGCACCA | TTTGGCCACAGGTGAGTT |
| *Eef1b2* | CTAGCAGTGTAGAAGACACCAC | CCTCACTTTCCTCCTCATCATC |
| *eEF2* | CTGGATGCAGACGACAAAGA | CTGTGACAGGAGAAGGAAGATG |
| *eIF2α* | GGACACGGGCGATGATTT | TCGGAGAACTCGGTGTAGTAG |
| *Gadd45b1* | AGATCCACTTCACGCTCATC | GGAGGTCTCTTGGTTCGTTAG |
| *Gpx1* | CGACATTGCCTGGAACTTTG | GGACAGCAGGGTTTCTATGT |
| *Gstp1* | ACGTACTTTCCGGTCAAAGG | AGAGACAGCTGCCTTTCTTATC |
| *Hmox1* | AGAGCAGCAGAAACAGGAAG | CCCTGTGATGCTCAGGATTT |
| *Nqo1* | GAAGACCGCACTGATTGTTTATG | TACAGATCCGACACTAGGACTT |
| *Prdx1* | TCTGTCATCTTGCCTGGATAAA | TCTTCCTTCAGCACACCATAG |
| *Cu/Zn-SOD* | CCATGGTGATCCATGAGAAAGA | TCACTGAGCGATGCCTATAAC |
| *Mn-SOD* | TTTAACGGTGGTGGCCATATTA | CCGACATCTTCTCCTTCATCTTC |
| *Hspa8b* | CAACACTACCATTCCAACCAAAC | TTGCTCTTTCGCCCTCATAC |
| *Hspa14* | GCTTCGAGACTTGGAACCTAAA | CTTTCTATGGTAACGGCCTCAG |
| *Hspa9* | CGAGTGATCAATGAGCCTACAG | TCAAAGGTTCCACCACCTAAAT |
| *β-actin* | CCATGTACGTTGCCATCCAG | CGCAAGACTCCATACCCAA |
