# Supplementary material for: Transcriptome analysis provides insights into copper toxicology in piebald naked carp (Gymnocypris eckloni)
Source: BMC Genomics. 2021 Jun 5;22:416. doi: 10.1186/s12864-021-07673-4 (PMC8178853; doi:10.1186/s12864-021-07673-4)
Supplement: Supplementary file 5 — Additional file 5: Table S2. Numbers of transcripts and genes in published papers. [file 12864_2021_7673_MOESM5_ESM.docx]

Table S2 Numbers of transcripts and genes in published papers.

| Species | NO. of transcripts | NO. of genes | Journal name | Published year | Reference link |
| --- | --- | --- | --- | --- | --- |
| *Hucho taimen* | 242,069 | 190,478 | Ecology and Evolution | 2017 | https://pubmed.ncbi.nlm.nih.gov/29375797/ |
| *Salvelinus alpinus* | 581,474 | 449,681 | PeerJ | 2018 | https://pubmed.ncbi.nlm.nih.gov/29441236/ |
| *Gymnocypris eckloni* | 551,430 | 337,481 | Frontiers in Physiology | 2018 | https://pubmed.ncbi.nlm.nih.gov/30298021/ |
| *Carassius auratus gibelio* | Not available | 242,341 | Fish and Shellfish Immunology | 2019 | https://pubmed.ncbi.nlm.nih.gov/31476388/ |
| Caretta caretta | 382,294 | 302,293 | Molecular Ecology | 2017 | https://pubmed.ncbi.nlm.nih.gov/28267875/ |
| *Dromiciops gliroides* | 507,815 | 31,438 | Molecular Ecology | 2018 | https://pubmed.ncbi.nlm.nih.gov/30240506/ |
| *Cheirogaleus crossleyi* | 543,950 | 15,768 | Molecular Ecology | 2018 | https://pubmed.ncbi.nlm.nih.gov/29319912/ |
